# *Campylobacter*, a zoonotic pathogen of global importance: Prevalence and risk factors in the fast-evolving chicken meat system of Nairobi, Kenya

**DOI:** 10.1371/journal.pntd.0006658

**Published:** 2018-08-13

**Authors:** Maud Carron, Yu-Mei Chang, Kelvin Momanyi, James Akoko, John Kiiru, Judy Bettridge, Gemma Chaloner, Jonathan Rushton, Sarah O’Brien, Nicola Williams, Eric M. Fèvre, Barbara Häsler

**Affiliations:** 1 Royal Veterinary College (RVC), Pathobiology and Population Sciences, Hatfield, United Kingdom; 2 Leverhulme Centre for Integrative Research on Agriculture and Health (LCIRAH), London, United Kingdom; 3 International Livestock Research Institute (ILRI), Animal and Human Health Programme, Nairobi, Kenya; 4 Centre for Microbiology Research, Kenya Medical Research Institute (KEMRI), Nairobi, Kenya; 5 Institute of Infection and Global Health, University of Liverpool, Leahurst Campus, Neston, United Kingdom; Swiss Tropical and Public Health Institute, SWITZERLAND

## Abstract

Campylobacteriosis is a leading foodborne zoonosis worldwide, and is frequently associated with handling and consumption of poultry meat. Various studies indicate that *Campylobacter* causes a substantial human disease burden in low to middle-income countries, but data regarding the organism’s epidemiology in countries like Kenya are scarce. In sub-Saharan Africa, 3.8 million deaths of children under-5 years of age are reported annually. Of those, 25% are caused by diarrheal diseases, and *Campylobacter* is one of the most frequently isolated bacteria from diarrheic children. With the growth of urban conglomerates, such as Kenya’s capital, Nairobi, changes in diets, food production systems, and retailing dynamics, it is likely that exposure and susceptibility to this pathogen will change. Therefore, the importance of *Campylobacter* disease burden in Kenya may increase further. The objectives of this study were: 1) to determine the prevalence of *Campylobacter* spp. in Nairobi’s small-scale chicken farms and meat retailers, and 2) to identify potential risk factors associated with its presence in those sites. The prevalence data provides the first detailed baseline for this pathogen in the urban Kenyan context. The risk factors provide context-specific insights for disease managers. A cross-sectional study of broiler, indigenous chicken farms, and chicken meat retailers, was conducted in a peri-urban, low to middle-income area (Dagoretti), and a very-low income informal settlement (Kibera) of Nairobi. Chicken faeces were collected using one pair of boot socks per farm, and 3 raw chicken meat samples were purchased per retailer. Samples were cultured for viable *Campylobacter* spp. using mCCDA, followed by blood agar plates in aerobic/microaerobic conditions for prevalence calculations. A questionnaire-based survey on sanitary, sourcing and selling practices was conducted at each site for risk factor identification using logistic regression analyses. A total of 171 farm premises and 53 retailers were sampled and interviewed. The prevalence results for *Campylobacter* spp. were between 33 to 44% for broiler and indigenous chicken farms, 60% and 64% for retailers, in Dagoretti and Kibera, respectively. Univariable logistic regression showed an association between *Campylobacter* spp. presence and the easiness of cleaning the display material used by the retailer. Restricting access to the flock was also associated with the pathogen’s presence. Multivariable logistic regression identified the selling of defrosted meat as a retailer risk factor (OR: 4.69; 95% CI: 1.31–19.97), calling for more investigation of the reported repetitive freezing-thawing processes and cold chain improvement options. At the farm-level, having a pen floor of material not easy to clean was found to increase the risk (OR: 2.31; 95%CI: 1.06–5.37). The relatively high prevalence of *Campylobacter* spp. across different areas and value chain nodes indicates a clear human exposure risk. The open nature of both small-scale broiler and indigenous chicken production practices with low biosecurity, hygiene and informal transactions, likely plays a role in this. While gradual improvement of farm biosecurity is recommended, risk factors identified suggest that consumer education and enforcement of basic food safety principles at the retailer end of the food continuum represent key targets for risk reduction in informal settings.

## Author summary

Gastrointestinal disease following food-poisoning can cause severe clinical signs in humans and represent high costs for society. Examples of bacteria causing foodborne diseases include *Salmonella* and *Campylobacter*. In low to middle income countries, where resources are limited and a significant part of the population cannot always afford treatment, foodborne diseases such as Campylobacteriosis can play an important role in child mortality. Chickens and undercooked chicken meat have been found to commonly harbour this bacterium. In countries like Kenya, where fast urbanisation is occurring and chicken farming systems are intensifying, diets and food retailing infrastructure are also changing. Scientific research has not yet well documented how widely distributed *Campylobacter* is in such changing contexts, and which risk factors can favour its presence. In this study, the researchers have investigated small chicken farms and chicken meat sellers in Nairobi, Kenya’s capital, to better understand the risk that Campylobacter could represent for human health.

## Introduction

Campylobacteriosis is one of the leading bacterial foodborne zoonosis globally [1], with handling and consumption of chicken meat identified as a major risk factor in high-income countries [2]. The estimated public health impact of *Campylobacter*-induced enteric disease is around 0.35 million disability-adjusted life years per year for EU-27, with annual costs estimated at about 2.4 billion euros [3]. Despite intensive research on the pathogen and testing of a range of control measures, campylobacteriosis has been the most frequently reported gastronintestinal disease in Europe since 2005 [4,5]. An overall rate of 59.8 cases of campylobacteriosis per 100,000 population was reported in 2014 for the European Union and two European Economic Area countries, ranging from 1.3 to 197.4 by country [4]. While differences in reporting, climatic conditions, and chicken production systems may explain differences in incidence, the epidemiology of the bacteria remains poorly understood and other factors may be involved. In low and middle-income countries (LMIC), surveillance for *Campylobacter* seldom exists in people and chickens, and data regarding the organism’s presence, risk factors and impacts are scarce. Yet, the disease burden of *Campylobacter* in the global South should not be underestimated. In Sub-Saharan Africa alone, 3.8 million deaths in children under 5 years are reported annually, 25% of which are caused by diarrheal diseases [6]. This bacterium is among the most common pathogens found in diarrheic children in LMIC [7]. In a multisite birth cohort study from 2009 to 2012 (MAL-ED study) in Asian, Latin American and African countries, *Campylobacter* spp. were the most frequently detected pathogens, occurring in 84.9% of 1892 children, and contributed the highest burden of diarrhoea in the first year of life. *Campylobacter* infection in children was associated with growth deficits across sites [8,9]. A *Campylobacter* isolation rate of 8% was reported in all-age diarrheic patients in Ethiopia [10], compared to 12% (higher than for *Salmonella* and *Shigella*) in Kenya [11]. A *Campylobacter* prevalence of 19% was reported in children under 5 from Morogoro, Tanzania [12], whereas a study in Western Kenya health centers isolated *Campylobacter* spp. from 42% of diarrheic children under 5 [13]. Hence, a better understanding of the sources of *Campylobacter* is needed to reduce diarrhoea-related child mortality. With the aim to address these data gaps, this study focused on Nairobi, Kenya, to investigate the epidemiology of *Campylobacter* spp. in a likely source, namely the chicken meat production system, in this setting.

Kenya’s capital, Nairobi, illustrates the global trend of fast urbanisation in LMIC countries. The human population has grown from 350,000 in 1962 to 3,375,000 in 2009, whilst the spread of informal settlements has led to over 60% of city’s population residing in conditions of significant poverty [14]. In parallel, the middle-class has been growing rapidly, with increasing demands in terms of food quality, and a surge in supermarkets and fast food outlets [15]. To meet the increasing demand in poultry meat, poultry production systems have been intensifying in Kenya [16]. An increase in commercial chicken farming, generally using imported fast growing broiler breeds such as the Cobb 500, is observed in and around Kenyan urban centres such as Nairobi, Mombasa, Nakuru, Kisumu and Nyeri, where the demand for poultry meat and market access for chicken producers are greater in comparison to rural areas. Outside of urban areas, indigenous chickens (i.e. local breeds which grow slowly and are used for egg and meat production) are the main chicken species kept [17]. These changes in retailing dynamics, diets, and poultry production systems, are altering the epidemiological setting for campylobacteriosis. While indications of protective immunity against the bacteria in adults [18] may have led to the disease been seen as low priority in LMIC, the evolving Nairobi setting may lead to significant changes in exposure and susceptibility to the disease in the population, and calls for a better understanding of the pathogen’s epidemiology.

While poultry is recognised as a major source of *Campylobacter* spp. [2], western studies have identified consumption of poultry meat, undercooked red meat, raw milk, untreated water, contaminated raw foods like salads, contact with pets and farm animals, and international travels as risk factors for disease in humans [2,16,17]. Studies investigating the disease in the global South are sparse. In the MAL-ED study covering 8 low-resource sites in Asia, Latin America and Africa, factors associated with a reduced risk of *Campylobacter* detection in children regular surveillance stools included treatment of drinking water, exclusive breastfeeding, access to an improved latrine, and recent macrolide antibiotic use [8]. *C*. *jejuni* and *C*. *coli* have been isolated from chickens, goats and sheep in Nigeria and similarities between strains isolated in chickens and humans suggest that poultry is an important source of human campylobacteriosis [7]. Risk factors identified for *Campylobacter* infection in people include home slaughtering and eating undercooked meat in Cambodia [19], the presence of animals or uncovered garbage in the cooking area, and lack of piped water in Egypt [7], contact with animals and HIV infection in Burkina Faso [20], young age, consumption of chicken meat and prepared salad in Tanzania [21], and poor hygienic conditions in LMIC in general [7].

The most important source of *Campylobacter* infection in chickens is thought to be the external environment [2]. Risk factors for *Campylobacter* presence reported for intensive commercial production systems in high-income countries include the use of contaminated water [22]; flock thinning (partial depopulation), carry-over from a previous flock following inadequate cleaning and disinfection, increasing bird age at slaughter and number of birds reared per year on farm [23,19]; organic rearing [24], broiler houses older than 15 years old, and long downtime between flocks [25]. A 2004 study in Senegalese broiler chickens found a 63% *Campylobacter* prevalence. On-farm presence of laying hens, cattle and sheep, lack of exclusive clothing for poultry workers, and use of chick transport cartons as feeders were found to increase the risk of infection in chickens, whereas thorough cleaning and disinfection of the poultry house were protective [26]. Two studies in South African broiler flocks found *Campylobacter* prevalence in chickens to be higher in rural areas (68%), compared to commercial indoor broiler flocks (47%) or layer flocks and (94%)[27]. To the authors’ knowledge, only one risk factor study has been published so far for chicken meat production systems in Nairobi, Kenya, which identified cleaning of the poultry house before restocking as a risk factor [28].

To mitigate carcass contamination by the intestinal tract of positive birds during the slaughter process [29], industrial slaughter and processing facilities in high-income countries use a variety of strategies such as chemical treatment, irradiation or freezing of carcasses to reduce the bacterial count [2,25]. At retailer level, general hygiene measures to prevent cross-contamination between the meat, retailer’s hands, contact surfaces and utensils, are recommended to minimise *Campylobacter* spread [30]. National prevalence in chickens and chicken meat have been found to vary greatly worldwide, from 4.9 to 100% in EU broiler carcasses [31], with a mean prevalence in broiler meat across Europe in 2015 of 46.7% [5], and from 8 to 100% in poultry meat at retail level across 32 different countries globally [32]. The mean prevalence reported for poultry meat in Senegal and South Africa in the latter study was 73.1%. In the sub- Saharan Africa context, *Campylobacter* prevalence in chicken meat was found to be 81.9% in poultry processing plants [33] and 100% in retail outlets in Nigeria [34], and varied between 11.1% and 100% in South African supermarkets [35]. One study found a prevalence for thermophilic Campylobacters of 77% (*C*. *jejuni* 59%, *C*. *coli* 39% and *C*. *laridis* 2%) in raw chicken sourced from butcheries, markets and supermarkets in Nairobi, Kenya [11], while studies in Ghana and Ethiopia found a prevalence close to 22% [36,37].

Risk factors identified for industrial commercial chicken production in high-income countries are highly context-specific and cannot be applied directly to informal meat production systems, such as small-scale Nairobi chicken farms, where biosecurity is limited, even in commercial broiler operations. Except for a few large integrated broiler companies and high-end supermarkets chains, informal production and retailing still dominate [31,14]. The lack of literature on *Campylobacter* risk factors in food animals and food animal products in LMIC, where rearing systems and level of hygiene may differ greatly from Western settings, represents a major gap [38].

Considering the public health importance of *Campylobacte*r, especially for vulnerable groups in LMIC, poultry’s predominant role in the global North as a risk factor, and the scarcity of epidemiological data in the context of rapid African urbanisation, the objectives of this study were: 1) to determine the prevalence of *Campylobacter* spp. in Nairobi’s small-scale chicken farms and chicken meat retailers, and 2) to identify potential risk factors associated with the presence of *Campylobacter spp*. in those same sites. The data provide a system-wide picture of the risks of exposure to *Campylobacter* at farm and retailer levels, and the first detailed baseline for this pathogen in the urban Kenyan context, whilst the identified risk factors help understand its epidemiology and provide insights for Kenyan disease managers.

## Methods

### Study design and study sites

The selection of Nairobi was based on the following criteria: representativeness of growing urban centers in East Africa, transitioning urban landscape and evolving chicken production systems. Nairobi, one of the major fast-growing urban centers in East Africa, with both a growing middle class and expanding informal settlements, is a prime candidate to investigate the epidemiology of *Campylobacter* in the context of transitioning urban landscape and chicken production systems. The study design was informed by previous work on the chicken meat value chains in Nairobi [39]. In the latter study, small-scale broiler and indigenous chicken farms, and small-scale broiler meat retailers were identified as key nodes, and were therefore targeted for the understanding of the risk of exposure to *Campylobacter*. Small-scale chicken farms were defined as a flock of 2 to 100 birds for indigenous chicken flocks, and 2 to 800 birds for broiler flocks, and small-scale broiler meat retailers were defined as any premise selling raw broiler meat (butchery) or a mix of raw and cooked meat (combination of butchery and small restaurant), not belonging to a franchise; they were found to be the most numerous chicken meat value chain actors in Nairobi. Poultry abattoirs and indigenous chicken meat retailers were found to be rare in Nairobi (indigenous chickens are commonly sold on-farm directly to consumers), and were therefore excluded. Large integrated broiler companies could not be sampled due to the sensitivity of the business information. A cross-sectional survey of small-scale broiler and indigenous chicken farms as well as broiler meat retailers was conducted (layer chickens were excluded). In order to provide a representative picture of Nairobi’s food system and major types of urban landscape found in the city, two areas of different wealth levels and production systems were purposely selected. Dagoretti, a low to middle-income, peri-urban area, characterised by a rural-like landscape with pockets of residential areas and moderate population density, was selected as a major livestock raising area within Nairobi, and due to its easy accessibility for the research team. Kibera, characterised by high population density, fully urban landscape and lower livestock activity, was selected as it represented the largest very low-income informal settlement (slum) in Nairobi. As major differences in the value chain structure and risky practices had been identified by Carron et al., 2017 [39], these two areas were targeted to test the hypothesis whether socio-economic status could affect the presence and survival of *Campylobacter* at farm and retailer level.

Sample sizes were calculated for independent populations (see S1 Appendix for more information on sample size calculation), namely two types of chicken production systems per area, and one retailer group per area, using an expected *Campylobacter* spp. prevalence of 50%, a 10% confidence limit and 90% confidence interval. No regular records of farms were available to guide the selection of farms to be sampled. The team worked with community elders that had been recruited to participate in the project to create a census of all broiler farms in each area. Since the number of broiler farms was limited (close to or below the calculated sample size), all were targeted for sampling. Because it is a common practice in the study sites to own indigenous chickens, it was not realistic to undertake a census of indigenous chicken farms. This resulted in an overall sample size for Dagoretti and Kibera, respectively, of 42 and 8 small-scale broiler farms, 67 and 63 small-scale indigenous chicken farms, and 21 to 40 small-scale broiler meat retailers per area. Using the target sample size for indigenous farms, a corresponding number of random GPS coordinates within each area was computer-generated using ArcGIS. The first farm found North of each GPS point by the sampling team was targeted for sampling. A census approach was used for broiler meat retailers, as these were reported by the elders to be few, and located along a few main streets in each area. In Dagoretti, the census of all butcheries selling chicken meat was performed by walking or driving along the main streets and asking employees whether they sold chicken. GPS coordinates for each retailer selling chicken were recorded. Due to the small number of retailers (close to or below the calculated sample size), it was decided to sample all retailers willing to participate. In Kibera, due to security issues, a key informant was asked to perform a similar retailer census with support from the local elders.

### Data collection

On chicken farms, one sample each of chicken faeces and/or housing litter was collected using boot socks dampened with sterile saline [40]. Three meat samples (100g or more) from different chicken carcasses were bought from each retailer. Each boot sock pair or meat sample was put in a sterile ziplock bag and stored in a cool box with ice packs, until testing at the laboratory within 5 hours of collection. All samples were cultured for viable *Campylobacter spp*, using a protocol based on the ENIGMA consortium 2017 study [40]. Boot sock samples were enriched using 50 ml of Exeter broth and incubated at 42°C under aerobic conditions with a minimal air space for 24 hours before sub-culturing. A 50g piece of each meat sample was cut aseptically, added to 200ml of saline and subject to stomaching for 1 minute; 5ml of the stomacher content was added to 5ml of double strength Exeter broth, and 10ml of the enriched sample incubated similarly to boot sock samples. All samples were then cultured for viable *Campylobacter spp*. at the Kenya Medical Research Institute (KEMRI) of Nairobi. Samples were first plated onto mCCDA and incubated for 48 hours at 42°C under microaerobic conditions (using CampyGen microaerobic gas pack in jars). Plates were visually examined for suspect *Campylobacter* colonies using colony size, shape and surface colour, and other key characteristic: *C*. *jejuni* 2.0–3.0 mm, flat/entire/glossy, grey/white, can be efflorescent (spreading moist), and *C*. *coli* 1.0–2.5 mm, convex/entire/glossy, creamy grey moist. For each mCCDA plate that showed growth for *Campylobacter* spp., four suspect colonies were subcultured on two different Columbia blood agar plates. One plate was incubated under microaerobic conditions for 48 hours at 42°C, and the other under aerobic conditions at 37°C for 48 hours. Growth in microaerobic conditions only was considered as positive for *Campylobacter* spp. A subset of isolates (428/560) was confirmed by LPX-PCR [41].

In order to evaluate risk factors for *Campylobacter* spp. exposure, a questionnaire was used to collect data from each site visited. The farmers’ and retailers’ questionnaire (S2 and S3 Appendices) covered the following categories of variables/themes (Table 1): 1) Farm or retailer’s environment and characteristics, 2) Management practices, 3) Biosecurity, health or sanitary practices, and 4) Sourcing and selling of chickens/chicken products. Questionnaires were written in English and conducted using Open Data Kit (ODK, https://opendatakit.org/about/tools/) software on electronic tablets. Sites and samples were identified by scanning unique barcodes. Enumerators were Kenyan citizens familiar with the city, bilingual in English and Kiswahili. Pre-sampling training of the enumerators on the questionnaires took place.

**Table 1:** Themes covered in the farm and retailer survey questionnaires and corresponding categories/sub-categories of variables included in the farm and retailer risk factor analyses (RFA) for the presence of *Campylobacter spp*.

	Farm questionnaire and RFA	Retailer questionnaire and RFA
Variable category	Variable sub-categories and examples	Variable sub-categories and examples
**Farm or retailer’s environment/characteristics**	Area Type of chickens and livestock on-site Water source used for chickens Type of housing (materials) Number of employees Total number of birds on-site	Area Number of employees Type of meat sold (chicken and non-chicken) and parts sold State of meat sold (frozen, fresh, defrosted)
**Management practices**	Type of confinement (scavenging, indoor exclusively, indoor part-time) Type of feed for chickens Disposal methods for/use of dead birds, manure Number of broiler batches on-site/raised per year Mixing birds of different source, thinning On-farm slaughter versus transport of live birds	Use of display area / display material, mixing of chicken parts-different meats Use of chilling devices/ their nature Cutting area/material On-site slaughter versus meat supply Storing practices (duration, cold chain device) Type of meat packaging
**Biosecurity/health or sanitary practices**	Use of antibiotics- type, source Biosecurity measures (restricted access to flock, foot bath, dedicated clothing) Cleaning and disinfection method/frequency of pen/ slaughter site Contact with other animals	Cleaning and disinfection methods/frequency for display area, cutting area Food safety accreditation Presence of flies on premises, ventilation, rodent control Handling money and meat, wearing gloves Cleaning method for hands/frequency
**Sourcing/ selling of chicken and chicken products**	Source of chicks Selling age, numbers sold	Nature and number of suppliers, frequency of supply Quantities bought (supply), sold, unsold

### Ethical approvals and participant consent

Prior to data collection, ethical approvals were sought from the ILRI-IREC (International Livestock Research Institute—Institutional Ethical Research Committee, project reference ILRI-IREC2016-01). ILRI-IREC is accredited by the National Commission for Science, Technology and Innovation (NACOSTI) in Kenya. Approval from the Royal Veterinary College (RVC) ethical committee was also received (project reference: URN 2015 1453). Permission to interview people was obtained from the Ministry of Agriculture and the local Veterinary Authorities. The study’s objectives and participants’ rights were explained in Kiswahili to farmers and retailers upon arrival at the site. Verbal and written consent to participate in the study were obtained before initiating data collection.

### Data cleaning and recoding

Variables in the survey data with too many missing observations (>25%) and variables with no substantial variability (>95% responses identical) were not kept for analysis. This first variable screening lead to a total of 45 farm exposure variables and 43 retailer variables for inclusion in the risk factor analysis. Using Excel and R version 3.3.2 (2016-10-31), each site (farm or retailer) barcode and meta data was linked to the corresponding sample barcodes and laboratory results. Inconsistencies and data gaps were reviewed with the field coordinator and discussed with laboratory technician to clean the database.

### Data analysis—Prevalence estimation

Using a chicken farm or retailer as a sampling unit, a site with one or more positive samples on culture classified as positive for *Campylobacter* spp. A sample was considered positive if at least one isolate was obtained and comfirmed by culture. Culture prevalences were calculated using QuickCalcs (GraphPad, http://www.graphpad.com). A Fisher’s exact test was used to compare prevalence between groups. The LPX-PCR results obtained for a subset of the samples were used to confirm the culture prevalences.

### Data analysis—Risk factors identification

A two-step statistical analysis using univariable logistic regression followed by multivariable logistic regression, was performed to identify risk factors for the presence of *Campylobacter* spp. at farm, or retailer level, respectively. No derivative analysis was made for farms or retailers from a specific area (Dagoretti or Kibera), or for a specific type of farm (broiler vs. indigenous chicken) due to the limited size of each subgroup. Rather, area and type of farms were included as confounders in the models.

A univariable analysis was performed in order to identify possible associations between the 88 selected exposure variables and the the presence of *Campylobacter* spp. using univariable logistic regression for each of the predictors. Odds ratio (OR) and 95% confidence intervals (CI) were calculated. All variables with a p-value (calculated with a likelihood ratio test) lower than 0.2 were retained for assessment in the multivariable analyses, except if the variable belonged to a nested question not applicable to the whole “farm” or “retailer” population.

Two multivariable logistic regression analyses were conducted independently, one for retailers, one for farmers. In each analysis, variables selected during the respective univariable analysis were included in the initial model. A stepwise backward selection procedure was used to refine models until all variables remaining in each model met the criterion of a p-value ≤0.05. Two-way interactions between predictors were assessed using a likelihood ratio test and considered significant if p ≤0.05.

In order to evaluate potential collinearity effect between predictors, the levels of association between risk factors identified during the univariable analysis were assessed using a Fisher test; risk factors with more than two-fold changes in the logistic regression coefficients were also checked during the selection process.

As data collection took place following a sampling frame designed for investigating *Campylobacter* spp. prevalence in two Nairobi areas and two types of chicken farms, multiple logistic regression models were built to account for the potential confounding effect of the study design. One farm model included “farm area” and “farm type” variables, despite their non-significance in the univariable analysis, whilst a second model did not include them. Similarly, one retailer model included the “retailer area”, and another did not include this variable.

The predicted probability was calculated for each observation based on the final model and the fit was assessed using the distribution of the model’s residuals, residuals close to zero suggesting a good fit [42]. Finally, an R-squared value was calculated [43]. All statistical analyses were performed using the statistical software R version 3.3.2 (2016-10-31).

## Results

Two broiler farms declined participating in the survey due to fear of pathogen introduction. Another 4 indigenous chicken farms declined sampling with no specified reasons. An estimated 25% of broiler farms on the census could either not be reached, or did not raise broilers over the course of the sampling months. In total, 171 farms were sampled; 18 and 7 small-scale broiler farms, and 78 and 68 small-scale indigenous chicken farms, in Dagoretti and Kibera, respectively. One questionnaire was administered per site, but as some sites had multiple sheds, 181 boot sock pairs were collected. An estimated 10% of small-scale broiler meat retailers declined participation in the survey, mainly due to the absence of the owner on the premises, or lack of time. A total of 53 retailers were successfully surveyed, 25 in Dagoretti, and 28 in Kibera; and 183 meat samples were collected.

### *Campylobacter* spp. prevalence

The culture prevalence of *Campylobacter* spp. in small-scale farms varied between 33 and 44% across types of production systems and areas, whereas the prevalence in retailers was 60% in Dagoretti and 64% in Kibera (Table 2). While *Campylobacter* spp. prevalence at retailer level was higher than at farm level, no statistically significant difference was found between the types of site. Out of the 429 isolates tested by LPX-PCR, only 1 was not confirmed as *Campylobacter*, suggesting reliable culture resuts. A total of 63% of the 428 *Campylobacter* isolates were *C*. *Jejuni*.

**Table 2:** *Campylobacter* spp. prevalence in chicken farms and chicken meat retailers in two areas of Nairobi.

Nairobi area	Type of site (farm/retailer)	Prevalence (90% confidence interval)
**Dagoretti**	Small-scale broiler farms	0.33 (0.16–0.55)
	Small-scale indigenous chicken farms	0.44 (0.34–0.54)
	Small-scale broiler meat retailers	0.6 (0.42–0.76)
**Kibera**	Small-scale broiler farms	0.43 (0.13–0.77)
	Small-scale indigenous chicken farms	0.37 (0.27–0.47)
	Small-scale broiler meat retailers	0.64 (0.47–0.79)

### Risk factor analysis—Retailers

#### Univariable analysis—Retailers

Table 3 summarises the variables in the retailer univariable analysis. Two variables were significantly (p<0.2) associated with a positive Campylobacter spp. culture: “selling defrosted meat” (p = 0.02), which increased the odds of Campylobacter spp. presence at the retailer premise by 4.69 (95% confidence interval, CI: 1.31–19.97), and a “display material not easy to clean” (OR: 7.86 (95% CI: 1.22–155.59]).

**Table 3:** Variables that showed evidence of association (p<0.2) with the presence of *Campylobacter spp*. in the retailer univariable analysis.

Explanatory Variable	Levels	Number of observations	*Campylobacter* frequency	P value <0.2 (Likelihood Ratio Test)	Odds Ratio (95% Confidence Interval)
**Selling beef**	Yes	11	9	0.13	3.24 (0.72–22.96)
	No	43	25		
**Selling defrosted meat**	Yes	19	15	**0.02**	**4.69 (1.31–19.97)**
	No	27	12		
**Number of carcasses bought per week for resale** *Reference*: *up to 50 carcasses/week*	Up to 50	32	22	0.15	*Reference*
	51–100	13	5		0.28 (0.07–1.06)
	>100	4	3		1.36 (0.15–29.48)
Easiness of cleaning the display material*	Easy to clean	24	14	**0.03**	*Reference*
	Not easy to clean	12	11		**7.86 (1.22–155.59)**
**Using hot water to clean the cutting equipment**	Yes	8	7	0.09	4.93 (0.78–96.07)
	No	46	27		

*“Easy to clean”: metal, plastic or tiles; “not easy to clean”: not metal, plastic, or tiles, usually wood or cardboard

#### Multivariable analysis—Retailers

Independent of inclusion of the “area variable” in the initial retailer model, the multivariable logistic regression analysis for retailers identified one risk factor, namely “selling defrosted meat” (p = 0.02), which increased the odds of a positive *Campylobacter* spp. culture by 4.69 (95% CI: 1.31–19.97). No signs of collinearity were observed in the stepwise backward variable selection process. The R-squared for the final model indicated that 12% of the variation in the data was explained by the risk factor “selling defrosted meat”.

### Risk factor analysis—Farmers

#### Univariable analysis—Farms

Table 4 summarises variables in the farm univariable analysis. Only three variables were found to have a significant (p<0.2) association with a positive Campylobacter culture from bootsocks: “restricting access to the flock” (p = 0.05), “cleaning method for pen” (p = 0.01), and “easiness to clean the pen floor” (p = 0.04). Restricting access to the flock was found to be protective (OR: 0.32; 95% CI: 0.09–0.90). Compared to a full cleaning (i.e. removing the litter, cleaning and disinfecting the pen), removing the litter only was found to increase the odds of Campylobacter spp. presence by 4.8 (95% CI: 1.67–17.46), while removing the litter and cleaning but without disinfecting increased the odds of Campylobacter spp. presence by 3.56 (95% CI: 1.14–13.69). A pen floor material not easy to clean, such as a wooden floor or one made of cardboards compared to a cement one, increased the odds of Campylobacter spp. presence by 2.31 (95% CI: 1.06–5.37).

**Table 4:** Variables that showed evidence of association (p<0.2) with the presence of *Campylobacter spp*. in the farm univariable analysis.

Explanatory variable	Levels	Number of observations	*Campylobacter* frequency	P value <0.2 (Likelihood Ratio Test)	Odds ratio (95% confidence interval)
**Total number of broilers on-site** *Reference*: no broiler**	No broiler	146	59	0.13	*Reference*
	1–250 broilers	13	7		1.72 (0.55–5.59)
	>250 broilers	12	2		0.29 (0.04–1.17)
**Have ruminants on the farm**	Yes	37	11	0.15	0.57 (0.25–1.23)
	No	134	57		
**Restrict access to the flock**	Yes	21	4	**0.03**	**0.32 (0.09–0.90)**
	No	150	64		
Number of broiler batches on-site***	1 batch	17	8	0.12	*Reference*
	2–4 batches	8	1		0.16 (0.01–1.19)
**Source of new birds**	Own breeding	115	46	0.12	*Reference*
	Nairobi farmer	13	7		1.69 (0.53–5.55)
	Agrovet	12	1		0.13 (0.01–0.71)
	Hatchery	13	7		1.69 (0.53–5.55)
	Market	5	2		0.96 (0.12–6.03)
	Upcountry (i.e. remote farm outside Nairobi)	13	4		0.64 (0.17–2.10)
**Cleaning method for pen** *Reference*: *full cleaning (defined as removing litter*, *cleaning and disinfecting the pen)*	Litter removed	90	43	**0.01**	Litter removed **4.80 (1.67–17.46)**
	Litter removed+ cleaned	47	19		Litter removed+ Cleaned **3.56 (1.14–13.69)**
	Litter removed+ cleaned+ disinfected	25	4		*Reference*
Practices thinning***	Yes	8	1	0.12	0.19 (0.01–1.51)
	No	14	6		
**Easiness of cleaning pen floor material**	Easy to clean	38	10	**0.03**	*Reference*
	Not easy to clean	124	56		**2.31 (1.06–5.37)**
**Easiness of cleaning pen wall material**	Easy to clean	54	18	0.19	*Reference*
	Not easy to clean	109	48		1.57 (0.80–3.15)
**Bedding type**	None	40	20	0.18	*Reference*
	Wood	67	25		0.60 (0.27–1.31)
	Earth	26	11		0.73 (0.27–1.98)
	Hay or mixed substrate	8	1		0.14 (0.01–0.91)

** Sites without broilers have indigenous chickens; other sites have broilers and a few indigenous birds

*** indicates questions only asked to broiler farmers; these variables were not included in the multivariable analysis.

#### Multivariable analysis—Farms

Initially, all variables identified in the univariable analysis were included in the model, as well as “farm type” and “area”, to account for the study design. However, “type of farm” showed collinearity with the “total number of broilers” variable, due to the “no broiler” level of this variable corresponding with “indigenous farm”. It was therefore decided to exclude the “type of farm” variable from the model, as the “total number of broilers” variable already accounted for the difference between indigenous chicken flocks and broiler flocks, as well as differences in broiler numbers on-farm.

The model was run twice, with and without the “area variable” to account for the study design. Either way, the only significant risk factor identified was “easiness to clean the pen floor material” (p = 0.03). A pen floor material not easy to clean was found to increase the odds of *Campylobacter spp*. presence by 2.31 (95%CI: 1.06–5.37).

The R-squared for the final model indicated that 2.6% of the variation in the data could be explained by the risk factor “easiness to clean the pen floor material”. Considering that only one risk factor was identified, the residuals obtained are acceptable. The low R-squared test result suggested that other factors not tested in the analysis (e.g. humidity, outside temperature) may influence the presence of *Campylobacter* spp. on-farm.

## Discussion

This study is the first to document *Campylobacter* spp. prevalence in both small-scale chicken farm and chicken meat retailer levels in Nairobi and to investigate factors determining the heterogeneity of *Campylobacter* presence in these settings. The results provide valuable insights into the potential risks of human exposure in an otherwise undocumented context. The great variability found in *Campylobacter* prevalence across broiler batches or carcasses in the EU, the limited number of similar studies in the East African context, and the differences in epidemiological units used in the literature (e.g. retailer versus carcass-level prevalence), make it difficult to compare these results directly with other studies. However, the relatively high *Campylobacter* prevalence results found in Nairobi retailers echoes some of the prevalence reported (73.1% or higher) in retail poultry meat in sub-Saharan Africa [28,36,37]. In Nairobi, Kenya, isolation rates of 59% for *C*. *jejuni*, *39% for C*. *coli*, *and 2% for C*. *laridis* were found in raw chicken sourced from butcheries, markets and supermarkets [11], with chicken meat tested less than 24 hours after slaughter showing a higher prevalence (85.3%). Time since slaughter might aso have influenced results in our study, since meat samples were not collected at the slaughter plant. Few studies identified much lower *Campylobacter* prevalence values, such as 21.7% in retail raw chicken meat tested in Ethiopia [36], and 21.9% of commercial chicken carcasses swabbed in Ghana [37]. Broiler flock prevalence in our study are moderately lower than in other sub-Saharan African studies (47% to 68% *Campylobacter* prevalence overall) [10,44], which might be due to the small number of broiler farms sampled, to a difference in size of commercial flocks, or a difference in sampling unit and testing methods. Few studies found a prevalence lower than 30%. A Ghanaian study found *Campylobacter* in 22.5% of ceacal samples [37], a Tanzanian study in 42.5% of chickens (various breeds) using cloalcal swabs [12] and an Ethiopian study, in 28.9% of chickens (various breeds) [45]. A study from 1988 found a prevalence of 51.5% in Kenyan broilers [46], whereas a 2018 study found an overall prevalence of 69.5% in Nairobi chickens [28]. The prevalence results in our study are indicative of a relatively uniform distribution of the pathogen across the chicken meat system studied. This can most likely be explained by the informal nature and overall lack of biosecurity in these systems, which is unlikely to limit the introduction of *Campylobacter* into either indigenous or broiler flocks. Unlike in Europe and North America, practices used in broiler versus “backyard” indigenous chicken farms in Nairobi share more similarity. Due to a lack of resources, small-scale Nairobi broiler rearing infrastructure is heterogeneous, using suboptimal materials, and often in proximity to other livestock. Flock management is often lead by irregular market access, with limited sanitary considerations. This is exacerbated in informal settlements where space is lacking, and resources are further limited. In such areas, a broiler flock can be found under a vegetable shop stall or staircase. The limited number of broiler farms observations, as fewer broiler farms than expected were identified, also limits the power of this study to identify differences between management systems. Indeed, studies in Ethiopia and Tanzania have identified marked differences in prevalence between broiler and indigenous chicken flocks, with conflicting results. Two studies in Tanzania found a higher *Campylobacter* prevalence in indigenous chickens (76.49% and 75%) compared to broilers (26.4% and 50%) [47,12]. Another Tanzanian study found no significant difference between broilers and indigenous chickens, but rather a higher prevalence in local chickens from rural areas compared to those in urban areas [48], while an Ethiopian study found significantly higher *Campylobacter* isolation rates in animals (chicken, sheep, cattle and pigs) in urban areas (56.7%) compared to rural areas (26.7%)[49]. Finally, a 2018 study found a prevalence of *Campylobacter* of 91.07% in broilers, 70.96% in layers, and 61.04% in indigenous chickens in peri-urban areas of Nairobi, Kenya [28]. The higher prevalence found in meat sellers compared to farms in our study may be explained by the risk of cross-contamination between chicken meat products of mixed sources during meat handling, cutting, storage and display. A Ugandan study found *Campylobacter* survived much better on wooden cutting boards than plastic or metal ones [50], wooden boards being widely used in Nairobi retailers. Nairobi-specific factors that may affect *Campylobacter*’s survival include the average temperature, which is constantly above 16°C, or the precipitation which is high (80 to 191 mm) during the two rainy seasons. Indeed, unlike the reported summer and autumn peaks of campylobacteriosis in Europe and North America, seasonality of *Campylobacter* has not been reported in LMIC, potentially due to a lack of study in tis setting [51]. The common practice of freezing and defrosting chicken meat in Nairobi, further discussed below, could also influence the bacteria’s presence. In addition to investigating the prevalence of *Campylobacter* in the meat system, determining the level of contamination of the chicken meat sampled in Nairobi retailers would have brought an additional key indication of the risk of human exposure, but was not feasible due to resource limitations. A higher load of *Campylobacter* on meat increases the risk of contamination of the direct meat environment and spread within a household, or site. The European Food Safety Authority (EFSA) has estimated a public health risk reduction of 50%–90% could be achieved, if all broiler batches complied with the critical limit of <1000 and <500 CFU/g of neck and breast skin, respectively [52]. However, the infectious dose for *Campylobacter* being low at a few hundred cells (500 or less) [53], prevalence of the bacteria at retailer-level was considered an appropriate indicator of the risk of exposure in this study.

Few explanatory variables were found to have a significant association with the presence of *Campylobacter* spp. in the univariable analysis or were identified as risk factors in the multivariable analysis. Retailers using a display material “not easy to clean”(e.g. made of wood or porous material) were shown to have higher odds of *Campylobacter* spp. presence, compared to those using a display material easy to clean. This is in line with literature describing lower levels of hygiene at retail-level as a risk factor [53]. A risk assessment of Campylobacteriosis linked to chicken meals prepared by households in Dakar, Senegal, determined that washing of cooking utensils during food preparation was not sufficient to significantly reduce the risk of Campylobacteriosis, whereas changing knife, board and dishes between pre and post-cooking was [54]. “Selling defrosted meat” increased the odds of *Campylobacter* spp. presence in both steps of the analysis. This finding is surprising given that freezing can be used as a strategy to reduce numbers of Campylobacters present on the meat [55,56,57,58]. However, freezing-thawing of chicken meat was found to be a common retailer practice in Nairobi and could favour re-contamination of the meat. Multiple retailers interviewed described how they turned off their freezer during the day to soften the meat for cutting, and turned it back on at night to preserve unsold meat until the next day. Freezing fresh chicken meat for 24 hours has been shown to reduce the log number of viable Campylobacters by up to 2.5 [55,56], and a 2–3 day freezing period to diminish the risk by 50–90% [52]. However, freezing temperatures in Nairobi are not verified, and incomplete freezing may be common. The repetitive freezing-thawing-refreezing practice observed in chicken retailers is driven by resource scarcity, and the demand from consumers for small quantities of chicken meat. The latter has led to a selling culture of cutting small pieces of meat from a whole carcass in the presence of the customer. Hardly any retailers were found to freeze small meat pieces in individual packaging. This may be due to customers wanting to see the carcass of origin. Where cold chain infrastructure is more affordable and food hygiene is strictly regulated and enforced, multiple freezing-thawing cycles are not allowed. Studies have found that the refrigeration prior to freezing, as well as the type of meat surface (e.g. skin versus meat muscle, or ground chicken) will affect the number of *Campylobacter* cells surviving freezing [32,36]. A 2013 study by the UK Food Standards Agency determined that the freezing temperature and length of time taken to freeze chicken livers influenced the bacteria’s survival [55]. Another study found lower *Campylobacter* prevalence in chicken meat from Malaysia wet markets compared to supermarkets, hypothesising that the chilling infrastructure in supermarkets favours survival of the bacteria whereas the ambient temperature of 29.6°C in wet markets is not favourable for growth [59]. On the other hand, a Kenyan study found lower levels of *E*. *coli* contamination in raw chicken meat sold in supermarkets compared to smaller-scale retailers [60]. This illustrates how specific freezing processes can influence risk reduction, and have a different effect depending on the pathogen. It highlights how significant the challenges linked to the cold chain can be in a context of limited resources, especially for a highly perishable product like chicken meat. Further research will need to investigate if the *Campylobacter* presence related to defrosted meat identified in this study originates from the freezing-thawing process with sub-optimal cold chain conditions, or from cross-contamination post-freezing. Since chicken meat freezing in Nairobi was well accepted by consumers, food safety interventions could capitalise on this practice and its potential for *Campylobacter* risk reduction. Awareness trainings regarding sanitary practices to avoid cross-contamination and promoting the freezing of small chicken pieces wrapped individually to minimise the handling and repetitive thawing-freezing could be considered.

In the farm univariable analysis, three variables were identified as having a significant association with the outcome of interest. The predictor “restricting access to the flock” was found protective. Arsenault et al. [61] specifically assessed the permanent locking of the broiler house, which was associated with a reduced risk of *Campylobacter* colonization in chickens. This practice is not readily applicable for indigenous free-ranging chickens. Even in the case of broilers, while greater access restriction could be encouraged by providing training to farmers, it is unlikely to result in any significant risk reduction without the general on-farm biosecurity being improved. This would require substantial investment, which in turn would demand external support or simultaneous improvement of small-producers’ market access and business profitability. Using a pen material “not easy to clean” and cleaning the pen without disinfectant were also found to increase the odds of *Campylobacter* presence, in line with literature citing inadequate cleaning and disinfection between flocks as risk factors [2]. Of interest, is a similar result from a 2018 Nairobi study, which only identified cleaning and disinfection of the chicken house before restocking as a risk factor (p<0.05) in the multivariable analysis [28].

Many of the risk factors identified in the literature for *Campylobacter* at farm-level (e.g. water source, thinning, biosecurity measures) [2,23,61,62], and retailer (or carcass) level (e.g. contact between different carcass parts (e.g. liver and meat), cross contamination via handling practices) [63], were tested in the univariable analysis, yet did not show any significant association with the presence of *Campylobacter*. The identification of few risk factors may be linked to the cross-sectional sampling design, less suited for risk factor analyses compared to case-control or cohort studies, and selected for the *Campylobacter* prevalence estimation objective. The limited number of observations, as well as the high number of potential risk factors, may have also limited the power of the study. In addition, the specificity of the Nairobi context and scarcity of similar studies in informal settings, are likely to explain some of the discrepancies with Western studies. The extreme variability in production practices and in the level of implementation of sanitary practices in this context is difficult to analyse accurately and illustrates the challenges of capturing risk factor data in messy settings. Overall, we can hypothesize that the minimal biosecurity and sanitary measures observed in small-scale Nairobi farms and retailers create an open system, with numerous sources of contamination, making individual risk factors hard to identify and isolate from the general environment. Still, the study, despite not following a risk assessment structure, provides useful risk indicators to be further investigated. While Roesel and Grace [15] have found that formal retailing settings in sub-Saharan Africa do not necessarily translate into a lower risk for consumers, repeating the analysis made for small-scale retailers in Nairobi’s high-end supermarkets, where stricter sanitary standards are applied, would enhance our understanding of the broad risk context. While the prevalence and risk factor analyses were designed to provide a system-wide picture of the risks of exposure to *Campylobacter* at farm and retailer levels, it should be noted that the lack of information regarding the origin of the carcasses at retailer-level limits our understanding of transmission dynamics in the chains. Indeed, retailers in Dagoretti have been found to source their carcasses locally, whereas Kibera retailers have reported selling low-value cuts from from major integrated broiler companies outside the informal settlement (see S4 Appendix for more information on the chicken meat supply).

In terms of recommendations arising from this study, the risk factors identified support training initiatives on biosecurity and food safety practices. Group feedback sessions are planned for farmers and retailer having participated in the study, including education on basic biosecurity principles, sanitation measures and safe handling of chicken meat. While gradual improvement of biosecurity measures (via appropriate cleaning and disinfection, better farming infrastructure and flock management) targeted at commercial farms should be supported, initiatives focusing on consumer education and enforcement of basic food safety principles seem more easily manageable, and with potentially greater impact as a first step, in informal settings. By using a risk-based sampling approach based on a value chain analysis to design the prevalence and risk factor analyses, this study presents methodological novelty. Substantial economic value chain studies in Africa can be found, but the combination of value chain analysis and risk identification, or disease investigation, remains limited. Finally, this study is the first to describe *Campylobacter* prevalence and risk factors both in chicken farms and chicken meat retailers at this level of detail in a peri-urban and informal settlement Kenyan setting, providing key insights into the specificities of *Campylobacter* epidemiology in quickly urbanising areas of East Africa.

## Supporting information

S1 Appendix Population sizes used in sample size calculation. (DOCX)

S2 Appendix Farmer survey questionnaire. (XLS)

S3 Appendix Retailer survey questionnaire. (XLS)

S4 Appendix Broiler meat supply per area. (DOCX)
