# Supplementary material for: Campylobacter, a zoonotic pathogen of global importance: Prevalence and risk factors in the fast-evolving chicken meat system of Nairobi, Kenya
Source: PLoS Negl Trop Dis. 2018 Aug 13;12(8):e0006658. doi: 10.1371/journal.pntd.0006658 (PMC6122836; doi:10.1371/journal.pntd.0006658)
Supplement: S1 Appendix — (DOCX) [file pntd.0006658.s001.docx]

**S1 A**ppe**ndix:**

**Table 1: Population sizes used in sample size calculation**

|  | **Dagoretti*- population sizes** | | **Kibera – population sizes** | |
| --- | --- | --- | --- | --- |
|  | broilers | indigenous | broilers | indigenous |
| **Small-scale chicken farms** | 111 farms  (33448 birds) | 6519 farms  (32598 birds) | 9 farms  (891 birds) | 831 farms (4159 birds) |
| **Small-scale chicken meat retailers** | Unknown** | NA | Unknown** | NA |
| **High-end supermarkets**  **(in or at periphery)** | 3 | NA | 3 | NA |

* Numbers are based on Nairobi 2009 livestock census (1) and livestock production officers’ focus group discussions which were organised in Nairobi for a related study (2). “Commercial birds” census numbers used for broiler numbers presumably include layer chickens too.

** No data on retailer numbers was found. The number of poultry butcheries/roadside vendors in Dagoretti and Kibera was discussed with local field experts and an estimated broad range was agreed upon: 30-100 of each retailer type in each area. Six supermarkets were found by driving in and around Kibera and Dagoretti; this total number was confirmed with key informants/village elders.

References:

1. GovKenya. Nairobi Livestock Population - Census 2009. Nairobi; 2010.

2. Alarcon P, Fevre EM, Muinde P, Murungi MK, Kiambi S, Akoko J, et al. Urban Livestock Keeping in the City of Nairobi: Diversity of Production Systems, Supply Chains, and Their Disease Management and Risks. Front Vet Sci. 2017;4(October).
