# Supplementary material for: Campylobacter, a zoonotic pathogen of global importance: Prevalence and risk factors in the fast-evolving chicken meat system of Nairobi, Kenya
Source: PLoS Negl Trop Dis. 2018 Aug 13;12(8):e0006658. doi: 10.1371/journal.pntd.0006658 (PMC6122836; doi:10.1371/journal.pntd.0006658)
Supplement: S4 Appendix — (DOCX) [file pntd.0006658.s004.docx]

**S4 Appendix:**

**Figure 1: Types of broiler meat suppliers used by retailers interviewed by area, based on survey questionnaire.**
